# Cultural practices, gender inequality and inconsistent condom use increase vulnerability to HIV infection: narratives from married and cohabiting women in rural communities in Mpumalanga province, South Africa

**DOI:** 10.1080/16549716.2017.1341597

**Published:** 2017-07-05

**Authors:** Sphiwe Madiba, Nomsa Ngwenya

**Affiliations:** ^a^ School of Public Health, Department of Environmental and Occupational Heath, Sefako Makgatho Health Sciences University, Pretoria, South Africa

**Keywords:** Gender and Health Inequality - intersections with other relevant axes of oppression, Sexual violence, HIV transmission, social norms, safe sex, gender roles, patriarchy

## Abstract

**Background**: Women in sub-Saharan Africa bear the brunt of the human immunodeficiency virus (HIV) epidemic, and older married women and those in cohabiting relationships are regarded as the largest HIV risk group. Although preventing HIV infection in married or stable relationships is an international HIV prevention priority, little is known about the influence of sociocultural contexts on safe-sex practice by women, particularly older women in rural communities in South Africa.

**Objectives**: This study aimed to examine how older women in a rural patriarchal society negotiate safer sex within marital and long-term cohabitation relationships, and their perceptions and experiences of barriers that influence condom use.

**Methods**: Focus group discussions were conducted with married and cohabiting women aged 40–60 years recruited from primary health facilities in a rural district in Mpumalanga province, South Africa. A thematic analysis approach was used to analyse the data.

**Results**: We found that although women reported negotiating safe sex in their relationships, they dreaded the possible consequences of suggesting condom use with their partners. Many factors made negotiating safe sex complex for these women: living in a patriarchal society where women play no part in sexual decision making, the fear of possible consequences of insisting on condom use, women’s inferior social position in marital relationships, cultural practices such as bride price, and gender inequality were the main barriers to practising safer sex.

**Conclusions**: Older married and cohabiting women dreaded negotiating safer sex in this patriarchal society where women’s subordination is legitimized. The findings suggest that the women were at high risk of HIV infection because of their inability to negotiate condom use, or to reject forced sex and non-consensual sex. There is a need for interventions targeting older married and cohabiting couples and key stakeholders within communities to create awareness about cultural practices and beliefs that undermine women and HIV prevention efforts.

## Background

The Joint United Nations Programme on HIV/AIDS (UNAIDS) estimates that 100,000 people in low- and middle-income countries aged 50 years or over acquire human immunodeficiency virus (HIV) every year. Consequently, HIV prevalence among those aged 50 years and older has been steadily rising. Of the people who acquire HIV every year, three-quarters (74%) live in sub-Saharan Africa (SSA), and people aged 50 years and over are a growing part of the HIV epidemic in the region. One unchanging feature of the epidemic in SSA has been the disproportionate gender impact, as women in SSA continue to bear the brunt of the epidemic relative to their male counterparts. In 2012, the national HIV survey in South Africa reported an HIV prevalence of 13% among people aged 50–54 years, and 12% among women and 6.9% among men aged 55–59 years [1].

A multitude of factors increase women’s vulnerability to HIV acquisition, including biological, behavioural, socioeconomic, cultural, and structural risks [2]. The dominant patriarchal culture and society in SSA exacerbates women’s inferiority, which affords them little or no power to protect themselves from HIV infection. In most patriarchal societies, the needs and desires of women are not considered and often they play no part in sexual decision making, nor are they allowed to express their sexuality [3]. While women in general continue to bear the brunt of the HIV epidemic, married older women, who were not regarded as vulnerable to HIV in past decades, are currently regarded as the largest HIV risk group. In most societies in SSA, HIV infection rates among married women are significantly higher than among single women, and over 80% of new HIV infections in women in SSA are estimated to occur in marriage or long-term relationships through heterosexual transmission [4,5]. This phenomenon could be attributed to the fact that condom use may be more acceptable to younger individuals than to their older counterparts; married individuals tend to be older than unmarried individuals [6].

The high number of cases of new HIV infection occurring among married and cohabiting couples poses a great public health challenge in SSA, as over 60% of the adult population is in marital relationships or cohabiting with regular partners [7]. This may explain the high prevalence of HIV among people aged 50 years and above. The risk of HIV in marriage and in stable or cohabiting relationships is due to an increased frequency of sex and low condom use [4]. In patriarchal societies, suggesting condom use or refusing unprotected sex in a marriage or cohabiting relationship is seen as questioning male authority [8]. In rural communities, patriarchal norms are entrenched and communities subscribe to strong cultural practices, beliefs, and traditional laws, which subordinate women. Traditional and cultural practices such as payment of Lobola (bride price) maintain the subordination of women in society [3,9,10]. Bride price involves payment in the form of cattle or money from the groom to the bride’s father and his family, and is part of the formal marriage in many African cultures [11]. This translates into a power imbalance in sexual interactions and increases the woman’s vulnerability to HIV, as negotiation for safer sex or refusal may result in violence [4,12]

While married women in general are vulnerable to HIV infection, married and cohabiting older women in rural communities are at significantly higher risk of HIV infection compared to their counterparts in urban communities. Women living in rural areas are marginalized and disempowered, and face geographical barriers in terms of accessing HIV knowledge and services [13]. In rural communities, the deeply entrenched beliefs about the role and position of women in society [3,4,11] place marriage as an important risk factor for HIV infection among women [4]. The perceived entitlement to sex that is ascribed to men by society and male dominance contribute to forced sex in marriage and increase the risk of HIV infection for women [14]. While the younger women may be in a position to question some of the gender norms in society because they have better educational qualifications and greater sexual freedom, older women are socialized into prescribed gender roles such as promising to honour and obey their husbands when they entered into marriage, which was likely to be with husbands considerably older than them [12]. The age difference between partners in patriarchal societies perpetuates the power imbalance in relationships, which increases the vulnerability of the women to HIV infection. Moreover, literature suggests that as men get older, their chances of becoming HIV positive increase [15].

UNAIDS proposes that preventing HIV infection in married or stable relationships be made an international HIV prevention priority [16]. However, in SSA, HIV prevention strategies continue to centre on young unmarried individuals despite increasing rates among married and cohabiting women [1,4,5]. The high prevalence of HIV infection among adolescents and young women in past decades resulted in HIV interventions resources dedicated to preventing HIV among adolescents and youth in urban settings. Consequently, little is known about the influence of the sociocultural contexts on safe sex practice by women, particularly older women in patriarchal rural communities in South Africa. Since married and cohabiting heterosexuals constitute an important population for HIV prevention efforts in SSA [17], a deeper understanding of the role that marriage plays in negotiating safer sex among older women is critical to effective HIV prevention efforts [4]. The aim of this study was to explore how older married and cohabiting rural women negotiate condoms and their perceptions of the sociocultural barriers that prevent the adoption of safer sex practices. It is essential that local sociocultural contexts of marriage, beliefs, and practices inform the HIV prevention strategies for different countries and settings.

## Methods

### Study design and setting

We used focus group discussions (FGDs) to collect data on married and cohabiting women between December 2014 and March 2015. This was a community-based study, which used primary health facilities to recruit married and cohabiting women who were aged between 40 and 60 years, through purposeful sampling. The women were recruited from two primary health facilities located at Enhlanzeni, a rural district in Mpumalanga province, South Africa. We opted for primary healthcare facilities to recruit the women because there are no defined community structures to facilitate the formation of FGDs. Mpumalanga province is one of the nine provinces in South Africa and is bordered by the countries of Mozambique and Swaziland. Although Enhlanzeni is a district in Mpumalanga province, the communities are predominantly of the Swazi ethnic group and have strong patriarchal norms similar to neighbouring Swaziland, with clearly defined gender roles for men and women. The communities subscribe to strong cultural practices and beliefs including polygamy, lobola (bride price), and traditional laws and cultural practices which subordinate women. Culturally, women are minors, and in these communities, minority is reinforced by marriage. Despite the high levels of HIV/acquired immunoeficiency syndrome (AIDS), women still have to negotiate with their spouses on the use of condoms [18,19].

### Data collection

We used FGDs to collect data because they provide a safe environment for participants to speak about sensitive information such as negotiating condom use in the marital relationship without personalizing the experiences. Condom use is a sensitive issue among elderly rural women, whose cultural practices and norms shun open discussions about sexual matters. The focus group also allows participants to speak in a specific context, within their specific culture, as opposed to expressing their individual views. However, because FGDs can be an empowering process for participants, the women provided both individual views and community perspectives of condom use [20].

The second author (moderator) and a research assistant trained in qualitative methods facilitated the FGDs in a culturally acceptable manner using the local language (Siswati). The moderator and research assistant are mature women who are of the Swazi ethnic group and understand the cultural context of the women. Since cultural practices and norms view discussions about sexual matters as inappropriate, the moderator played a role in ensuring that the women felt at ease by providing clear explanations of the purpose of the group and assured the women of the confidentiality of the discussions. The open-ended questions also allowed the women to express their views using concepts unique to their language. All FGDs were facilitated in a private room to allow the women to speak freely; they were recorded after obtaining consent from the women and each session lasted for about one hour. On average, each focus group comprised about seven women, making a total of 36 women. At the end of each FGD, the moderator collected background information including age, marital status, employment, and level of education.

### Data analysis

The moderator transcribed the audio-recorded FGDs verbatim, using the language of the interviews, and later translated the transcripts into English. The first author reviewed the data for accuracy by playing back the recordings while reading the translated transcripts. We used a thematic analysis approach to analyse the data, and the first step in analysis was to read and reread the transcripts to familiarize ourselves with the data. We then coded two transcripts independently and identified commonly emerging codes using line-by-line coding. Scheduled meetings during which we compared the independently generated codes and identified themes to develop an initial codebook followed the initial generation of codes. All the transcripts were subsequently imported into NVivo 10, qualitative data analysis software that was used to apply codes to all the transcript. We engaged in a rigorous process to define and reach consensus on the emerging themes and subthemes, which we use to present the findings.

We used several methodologies to ensure trustworthiness: we held peer debriefing sessions after each FGD, we used the local language to facilitate the sessions, extensive field and interview notes were taken, we transcribed the transcripts verbatim, and both authors took part in data analysis using NVivo qualitative software [21].

## Results

### Description of study sample

Five FGDs were conducted with 36 women aged between 40 and 60 years. Most (16 out of 36) of the women were married, while the rest (20 out of 36) were in long-standing cohabiting relationships. Three women had no formal education, 14 had primary education, 18 had secondary education, and only one had tertiary education. Most (29 out of 36) were unemployed and only seven had some form of employment.

### Themes

Eight major themes and four subthemes emerged from the analysis of five FGDs: (1) reluctance to use condoms; (2) reduced sexual pleasure; (3) fear (of physical violence, separation, financial dependence, and labelling); (4) cultural beliefs; (5) social position of men; (6) forced sex; (7) desire to control condom use; and (8) breaking the submissive role.

#### Reluctance to use condoms

The women reported that they were aware of their husbands’ extramarital activities and suggested condom use because they knew that condoms would prevent them from acquiring HIV. However, most reported that their partners refused to use condoms and associated the request to use a condom as suggesting that they were infected with HIV.

If you tell your husband about the condom, he wants to know where that is coming from, he would ask “what have you heard or what have you discovered, I am not sick; I don’t have the disease, why do you want me to wear a condom?” (Participant 2, FGD 1)

The man that I am staying with told me bluntly that he would not use a condom. Even after I tested positive during pregnancy in the year 2000. (Participant 2, FGD 4)

#### Reduced sexual pleasure

All the women in the focus groups believed that men generally disliked condoms and had negative attitudes towards their use. Despite the fact that they refused to use condoms, one of their reasons for refusal was the belief that condoms prevent sexual pleasure.

Men say they do not feel pleasure when they have the condom on. (Participant 7, FGD 1)

A man will tell you straight that you do not eat a sweet with a paper because you will not feel its taste. He wants to feel it flesh-to-flesh. (Participant 2, FGD 2)

I will not use a condom because my husband says he does not want a condom, and if I keep on insisting on a condom, he says he does not feel pleasure, and so …, I want to please him so that he gets what he wants. (Participant 4, FGD 1)

#### Fear

Four subthemes emerged that explained the fear that the women experienced when negotiating condom use: (i) physical violence; (ii) separation; (iii) financial dependence; and (iv) labelling.

##### Physical violence

Violence against women is a global problem and a critical driver of vulnerability to HIV infection among women in SSA. Violence against women is triggered by various factors; however, there is always a threat of potential violence when women take the initiative in sex, suggest condom use, or refuse sexual advances across population groups in SSA. While most women dreaded the possible consequences of suggesting condom use with their partners, some experienced sexual and physical violence from their husbands or cohabiting partners after suggesting condom use. This is what those who experienced violence had to say:

He does not agree to use a condom; he tells me straight and does not hide, you see …, He says it straight to my face and to the extent of beating me. (Participant 1, FGD 1)

He will just forcefully come into you and even tell you that “I want you to go and tell your people that I have done this to you because you are refusing me my food that I have paid for, isn’t I have paid lobola (bride price)”. (Participant 4, FGD 2)

##### Separation

While the fear of physical violence was a barrier for negotiating safe sex, the women were also fearful that asking for condom use might result in rejection and abandonment by their partners. They also believed that persistently asking that a condom be used might result in their husbands seeking sexual favours from other sexual partners, which created the fear of separation or divorce. In a patriarchal society where the marital status of women is revered, the thought of being deserted by their partners and the subsequent stigmatization by the community was a barrier to negotiating condom use. The women would often endure physical and sexual violence than face abandonment or divorce, as reflected in their statements.

So …, when he comes to you as his wife, he tells you …, he does not ask you. He will say, “I want you to do what I say”. So …, you end up thinking that if I do not do what my husband says, he will divorce me and I will be ridiculed in the community. (Participant 4, FGD 1)

If you suggest a condom, he will say it is better for you to pack your bags and go, and I will tell your people that you are refusing me sex. (Participant 4, FGD 2)

He will pretend as if it does not matter [asking for a condom] but will go to other women for sex and when you ask him about it he will say it is because you want a condom. So …, you end up agreeing to have sex without a condom. We are scared of being rejected. (Participant 8, FGD 5)

##### Financial dependence

Rural communities in South Africa are characterized by high rates of unemployment and poverty, which affects women and children to a large extent. Men are often migrant workers in the big cities and mines, leaving the rural women to look after the children. Most of the women in this study were unemployed and depended on their husbands for financial support. The dependence on their husbands made them afraid to ask for condoms because they feared that it would result in divorce, which would have undesirable ﬁnancial implications for them and their children.

The main problem is that women are dependent upon men and are scared to suggest condoms, and will continue to do so even if one is now sick [HIV infected]. (Participant 8, FGD 5)

The other thing is …, you now have children and you ask yourself “where will I go with such a lot of children?”, therefore you will allow him to do what he wants. (Participant 5, FGD 5)

##### Labelling

Societal norms about appropriate sexual behaviour for women restrain them from openly discussing condom use with their partners, particularly in marriage. Women who openly discuss or suggest condom use with their partners are likely to be perceived as overly interested in sex, promiscuous, or suspected of having another sexual partner. The women reported that their partners perceived them as promiscuous when they suggested using a condom.

When you tell him to put on a condom, he will tell you that you have started to sleep around, that you are now promiscuous. (Participant 2, FGD 5)

He will say you have an extramarital affair …, my husband once told me that after I requested him to use the condom. He said, “You are asking for a condom because you have another man”, he said it meant that when he is away at work, there are other men. (Participant 6, FGD 1)

#### Cultural beliefs

Cultural beliefs and practices such as lobola or bride price (where the bride’s family receives financial compensation from the potential husband) perpetuate the idea that a woman is her husband’s property and play a major role in the use of condoms in these settings. Men refused to use condoms because they had paid bride price. The narratives from the women suggested that men believed that they were entitled to sex because of the payment of bride price.

My husband says that he did not pay lobola (bride price) for a condom but for me, he does not agree to use a condom. (Participant 1, FGD 1)

If I ask him to use a condom, he says, “I have paid lobola (bride price) and I have married you, I will do as I wish”. I will agree to his rules because I am a woman. (Participant 6, FGD 2)

He will say, “I did not pay lobola (bride price) to have sex in a plastic, why did I pay lobola (bride price) if you have to give it to me in a plastic?” (Participant 5, FGD 5)

#### Social position of men

Societies characterized by male dominance and gender inequality create power imbalances within a relationship. While the man occupies a high social status in the marital relationship and in society, the woman occupies a subordinate and inferior position and plays no part in sexual decision making. The women reported that their partners felt that they could not be told by a woman when and how to have sex. As a result, the women could not negotiate condom use successfully. Moreover, the age of the husband or partner and the length of the relationship were barriers to successful negotiation for condom use.

The man will tell you that “I am old; I am not a child that you can tell me to use a condom”. What can you do if he does not want to use condoms because he is old and it is a long time since he has been staying with you? All of a sudden, you say he must use a condom. (Participant 5, FGD 2)

He will tell you that we now have four children and we were not using a condom, why are we now supposed to use a condom? (Participant 5, FGD 3)

#### Forced sex

Forced sex and sexual coercion are commonly reported violent practices in marriage and cohabiting relationships in SSA, and are critical drivers of HIV transmission. Forced sex in patriarchal societies is perpetuated by men’s perceived entitlement to sex because of payment of lobola or bride price. The women reported that sex was often forced and that their husbands used pressure, threats, and physical force to have sex against their will. On the other hand, some of the women believed that they had to please their husbands as a necessary part of marital duties. These women would not refuse sex even when sex was unprotected and their partners used force.

He will forcefully do it …, you will try to scream but he will continue until you leave him to finish. (Participant 4, FGD 2)

So he will rape you …, it is so common, to the extent of hitting you. (Participant 7, FGD 5)

Sometimes he does not rape you but you will agree to sex even though you are not willing. It does not mean that he has forced you but you agreed yet you did not like it, so it is similar to rape because you did not want to. You agree because you are scared that he will leave. (Participant 8, FGD 5)

I will not use a condom because my husband says he does not want a condom, and if I keep on using a condom, he says he does not feel pleasure, and so …, I want to please him so that he gets what he wants. (Participant 4, FGD 1)

#### Desire to control condom use

Most of the women wished to have control in condom use and desired to be taught how to use the female condom which, they believed, would solve the problem of involving the man in their self-protection.

I heard that there are female condoms but I do not know them but I wish to know them. (Participant 2, FGD 5)

We must use the female condoms because we cannot succeed to fight the men all the time because some are aggressive and will tell you that this is my house …, then, it is better that women be the ones that are using it [female condom]. (Participant 3, FGD 2)

#### Breaking the submissive role

While most women were unsuccessful in negotiating condom use, a few showed high levels of empowerment with regard to safer sex, which were influenced by the high prevalence of HIV/AIDS in their communities. Consequently, they refused unprotected sex and abstained from sex if the partner refused condom use and/or separated from their partners because of the man’s refusal to use a condom.

I separated from my man because he was refusing to use a condom. He asks me if I can eat a sweet from the plastic. I said to him that sex is not a sweet. I said, I [would] rather lose the marriage than die. (Participant 1, FGD 1)

He was already taking ARVs [antiretrovirals] and I was not, but he was refusing to use a condom, and because we did not understand each other I ended up running away, we separated. (Participant 2, FGD 1)

My husband works far from home and when he comes home and refuses the condom I tell him that if you are refusing a condom you are not getting anything. I really do not give him anything if he does not want the condom. When I say I do not want sex without a condom, he ends up taking it and using it because he wants to sleep with you. He will put the condom on and we will continue and that makes us safe. (Participant 6, FGD 2)

## Discussion

We examined how older women in a rural patriarchal society negotiate safer sex within marital and long-term cohabitating relationships, and their perceptions and experiences of barriers that influenced condom use. This study is embedded in a society with entrenched cultural practices and patriarchal systems where the minority status of women is reinforced through marriage. We found that although the women reported that they negotiated safe sex with their partners, they dreaded the possible consequences of suggesting condom use. Many factors made negotiating safe sex complex for these women, as they live in a patriarchal society where women are not allowed to express their sexuality and play no part in sexual decision making [3]. The men are often much older, have been in a marital relationship for a long time, and subscribe to cultural beliefs of men’s perceived entitlement to sex [14]; for them, openly discussing condom use with their female partners was seen a sign of lack of trust and suspicions of unfaithfulness. Of more importance, openly discussing condom use was seen as questioning male authority.

For the women, perceived consequences of requesting or insisting on condom use included the fear of rejection and abandonment by their partner, fear of separation or divorce, and fear that the partner would seek sexual favours from other women [9,11]. Unlike younger and/or unmarried women who can insist on condom use or refuse sex, older woman in marital and long-term cohabiting relationships do not have that option. The fear of abandonment or separation in communities where marital status is held in high regard affected their ability to negotiate safer sex to a large extent. They failed to persuade their husbands to use condoms despite knowing that the men had multiple sexual partners. Nyamhanga and Frumence refer to this phenomenon as compelled tolerance of high-risk sex in marriage [11]. The risk of HIV infection was especially heightened in this male-dominated patriarchal community where engaging in unprotected sex with multiple sexual partners in marriage was acceptable. The assumption that when the risk of HIV infection is known, married women would want to use condoms or abstain from sex [22] is only relevant to financially autonomous women who also have a higher level of education. The profile of the women in their study is different from that of the older women in the current study, who were unemployed and totally dependent on their husbands.

We found that for some of the women, attempts to insist on condom use and/or refusal of sex without condoms resulted in physical abuse and/or forced sex, which exposed the women to a high risk of HIV infection [11,14]. Women tolerated forced sex in submission to their husband or partner’s authority because they were dependent on their husbands. Economic powerlessness among rural older women is significant in view of the high levels of unemployment and poverty in these settings. Moreover, in a patriarchal society, the main axis of power is the legitimization of the subordination of women and dominance of men [17,23]. Mugweni and colleagues advocate for participatory research to investigate culturally acceptable ways of addressing female powerlessness within sexual relationships[14].

The powerlessness of women to negotiate condom use is also rooted in gender inequality. We found that although the women were 40 years old and above, gender inequality was perpetuated by the wide age difference in the marital relations. While a woman occupies an inferior social position in the marital relationship in a patriarchal society, when she enters into a marital relationship with a partner who is much older, she is also culturally bound to honour, obey, and submit to the authority of the man [9,11]. The wide age difference rendered the women voiceless in more ways than one; they were not heard or listened to when they raised the issue of HIV testing and prevention or practising safe sex. We found that societal expectations of obedience and tolerance forced the women to stay in marriages that increased their risk of acquiring HIV [11]. For these women, being unemployed, living in a male-dominated rural society, and being in the marital relationship for a long time exerted pressure to stay married.

Gender inequality was also significantly driven by the payment of lobola (bride price), which gave men entitlement to sex whenever they wanted and increased the vulnerability of women to sexual violence and HIV infection [9,11,24]. Women were subjected to constant reminders about bride price and men’s entitlement to sex. This rendered the women powerless to suggest and insist on continuous condom use [23]. However, some of the women were not completely powerless even in the face of bride price: they suggested condom use to point out their partner’s risky sexual behaviour in an effort to protect themselves from acquiring HIV. All the same, standing up for their rights was responded to with violence and forced sex. In a previous study, Nyamhanga and Frumence [11] cautioned that when the man’s expectation of entitlement to sex is not met, he may resort to violence and force the partner into submission to have non-consensual sex.

As mentioned, the women were not completely powerless; some actively participated in sexual decision making and were able to refuse unprotected sex and insist on continuous condom use. While a study conducted in Zimbabwe attributed the participation in sexual decision making to educational and economic independence [9], the older women in the current study were uneducated but motivated by the desire to protect themselves from HIV infection. Moreover, the active participation of women in sexual decision making was not limited to the few who insisted on condom use but included those who felt strongly about undergoing HIV testing together with the partners and openly discussed HIV- and AIDS-related matters. Despite a failure to successfully negotiate condom use, the women desired to have control in condom use, particularly the female condom, because its use does not require negotiation with the male partners. Although the need to control condom use is encouraging, Koster and colleagues [24] caution that promoting female condom use within marital and long-standing relationships requires the active involvement of men. Promoting the female condom as a female-initiated method is likely to be ineffective in settings where unequal gender relations prevail.

## Conclusions

We found that older married and cohabiting women dreaded negotiating safer sex in this patriarchal society where subordination of women is legitimized. The fear of possible consequences of insisting on condom use, the women’s inferior social position in the marital relationship, cultural practices such as bride price, and gender inequality were the main barriers to practising safer sex. The implications of these findings are that empowering married and cohabiting women in male-dominated societies to negotiate for safer sex calls for complex but culturally acceptable interventions. Given that safer sex in marital relationships is negotiated in a wider sociocultural context, this calls for effective sociocultural acceptable prevention strategies. It is imperative that interventions take into consideration how safe sex in marital relationships is constructed when developing messages to sensitize communities about practices that undermine HIV prevention efforts among the older generation. It is also crucial to develop culturally acceptable ways to position the bride price in a context where health providers, community stakeholders, men and women, and traditional leaders begin the discussions of the role of bride price in the context of the risk of HIV infection in older married women.

Suggesting that a condom be used resulted in verbal abuse, physical violence, and forced sex for some of the women, while others would not even think of suggesting condom use because they believed that their husbands were entitled to sex. It is evident that gender inequality compromised the efforts of women to protect themselves from acquiring HIV, which may explain why most of them were infected with HIV. Healthcare providers have a crucial role to play in opening up discussions and communication on safe sex with older men and married or cohabiting older women, a topic that is generally targeted at younger groups of people who are considered to be at high risk of transmitting HIV. Couple counselling with couple-based HIV prevention messages is one strategy that can empower men and women in marital and cohabiting relationships to have safe and healthy sexual relationships and prevent HIV transmission. The participation of men in HIV prevention activities is essential, particularly in a patriarchal society where male behaviour is one of the main determinants of HIV infection in women. Nevertheless, these strategies should be preceded by culturally sensitive community awareness programmes and education on condom use among the older generation.

The findings suggest that most of the women were at high risk of HIV infection and reinfections because of their inability to negotiate and insist on continuous condom use, and to refuse forced sex and unprotected non-consensual sex. The South African National Strategic Plan (NSP) on HIV and sexually transmitted infections acknowledges that in order to respond to the HIV epidemic, there is a need to implement interventions to address gender norms and gender-based violence, which drive the HIV epidemic. It is imperative that culturally acceptable interventions are developed to address the gender inequalities identified in sexual relationships in the study setting. These interventions should also aim to educate men and women about the rights of women in sexual decision making, to enhance the sexual health of women but also to empower men to understand the role of women in sexual decision making. The NSP also calls for the development of strategies to address the structural, social, economic, and behavioural factors that drive the HIV epidemic. Therefore, safer sex strategies that respond to the context of different population groups and communities are fundamental to the global HIV prevention interventions that address sexual transmission of HIV. While it may be ideal to develop interventions for men and women, equipping women with skills to use condoms and increase access to female condoms should be the focus of all interventions.

## Paper context

HIV prevention strategies and resources dedicated to preventing HIV continue to focus on adolescents and young women. Yet, HIV prevalence among those aged 50 years and older has been steadily rising, and married and cohabiting older women are the largest HIV risk group. However, little is known about safe sex practice by older women within marital and cohabiting relationships in rural communities. HIV prevention strategies should respond to the sociocultural contexts of marital relationships.
